# P151: National action plan for the local production of alcohol-based hand rub for hand hygiene in Senegalese hospitals: situational analysis

**DOI:** 10.1186/2047-2994-2-S1-P151

**Published:** 2013-06-20

**Authors:** D Faye, A Ndir, R Kande, B Ndoye

**Affiliations:** 1 Fann Hospital, Dakar, Senegal; 2 PRONALIN, Ministry of Health, Dakar, Senegal; 3 DPM, Dakar, Senegal

## Introduction

Hand hygiene is an important measure to reduce the risk of infections related to care proceedings. WHO proposes to use alcohol-based hand rubas a strategy for improving hand hygiene in health care settings. As part oftheimplementation of this strategy, Senegal has initiated anational action planfor the installation ofmanufacturing unitsin hospital pharmaciesand national medicinessuppliers.

## Objectives

The objective is to strengthen the capacity ofall pharmaciesin thelocal manufacturingof the product and to assess the capabilities of each structure to house a manufacturing unit.

## Methods

To install these units the Minister of Health signed a circular allowing us to do a situational analysis of pharmacies. The study was funded by Intrahealth.

## Results

We visited 39 pharmacies. In 35 pharmacies we found at least one pharmacist and a pharmacy technician in each structure. The premises were compliant production in 70% of cases. Only 12% of pharmacies had equipment.

## Conclusion

In Senegal is located one of the pilot hospital of the African Patient Safety Partnerships Program supported by the WHO for local production of alcohol-based hand rub.The experience of thishospitalis used toestablish apartnership between the Departmentof Health andIntrahealth, USAIDagencyinvolved inthe provision ofqualitycare.These results will be shared with the University Hospitals of Geneva.

## Disclosure of interest

None declared

